# Evaluation of the Molecular State of Piperine in Cyclodextrin Complexes by Near-Infrared Spectroscopy and Solid-State Fluorescence Measurements

**DOI:** 10.1155/2019/7530480

**Published:** 2019-02-11

**Authors:** Toshinari Ezawa, Yutaka Inoue, Isamu Murata, Koichi Takao, Yoshiaki Sugita, Ikuo Kanamoto

**Affiliations:** ^1^Laboratory of Drug Safety Management, Faculty of Pharmacy and Pharmaceutical Sciences, Josai University, 1-1 Keyakidai, Sakado-shi, Saitama 3500295, Japan; ^2^Laboratory of Bioorganic Chemistry, Faculty of Pharmacy and Pharmaceutical Sciences, Josai University, 1-1 Keyakidai, Sakado-shi, Saitama 3500295, Japan

## Abstract

The purpose of this study was to evaluate the physicochemical properties of piperine (PP) in ground mixtures (GMs) of PP with *α*-, *β*-, or *γ*-cyclodextrin (CD) under conditions of humidity, heat, and humidity-heat. In solid-state fluorescence measurements, the fluorescence maxima for GM (PP/*α*CD = 1/2), GM (PP/*β*CD = 1/1), and GM (PP/*γ*CD = 1/1) were observed at 463, 472, and 469 nm, respectively. On the other hand, the humidified GMs exhibited maxima at 454, 460, and 465 nm, while the humidified-heated samples displayed fluorescence maxima at 455, 455, and 469 nm, respectively. Therefore, the molecular behavior of PP with *α*, *β*, and *γ*CD was concluded to vary upon the coordination of water molecules. NIR and solid-state fluorescence measurements revealed that the molecular behavior of PP inside the *α*, *β*, and *γ*CD cavity changed by water and heat factors depends on the mobility of the methylenedioxyphenyl group.

## 1. Introduction

Pepper is a household spice widely used in food additives and seasonings. In addition, pepper is an important component of traditional medicine in Asian countries. In recent years, diet food has been attracting great attention due to the continuous increase in obese population. Also, the demand for pepper is increasing with the rapid development of seasonings [1]. Piperine (PP) is the major pungent ingredient in Piperaceae pepper (*Piper nigrum*). The chemical structure of PP is (2*E*,4*E*)-1-[1,3-benzodioxol-5-yl)-1-oxo-2,4-pentadienyl] piperidine. Among its properties, PP has been found to significantly reduce serum triglyceride, total cholesterol, low-density lipoprotein cholesterol, and very low-density lipoprotein cholesterol levels in high fat-fed rats [2]. PP significantly increases high density lipoprotein cholesterol levels and its beneficial effects have been reported to reduce dyslipidemia [3]. In addition, PP displays beneficial food functions such as nutrient absorption promotion and anti-inflammatory and antidepressant action [4–6]. As a major component of pepper, PP is expected to have diverse applications. The geometry of the conjugated diene of PP may present several isomeric forms upon* cis*-*trans* isomerization: isopiperine =* Z*,*E*-(*cis*-*trans*)-piperine, chavicine =* Z*,*Z*-(*cis*-*cis*)-piperine, and isochavicine =* E*,*Z*-(*trans*-*cis*)-piperine [7]. Changes in the amide group of PP influence the intensity of its physiological function [8].

Cyclodextrins (CDs) are polysaccharides composed of D-glucopyranose linked by *α*-1,4 glycoside bonds. CDs are classified as *α*, *β*, and *γ*CD according to the number of D-glucopyranose units (6 to 8). In the safety assessment by the FAO/WHO Joint Expert Committee on Food Additives (JECFA), the acceptable daily intake (ADI) was defined as ‘‘up to 5 mg/kg/day” for *β*CD. In the background review for cyclodextrins used as excipients (CPMP/463/00 Revi.1), the total daily dose for alpha and gamma CD is fixed to 6000 and 10000 mg/day, respectively. At high concentration, CDs can induce laxative effect due to osmotic activity. CDs present a hydrophobic cavity due to the ether group located inside cavity, while the outer ring is hydrophilic. CDs form inclusion complexes via hydrophobic interactions [9]. The secondary hydroxyl groups (C_2_-OH and C_3_-OH) of CDs are located on the broad annular rim, while the primary hydroxyl group (C_6_-OH) is located on the narrow annular rim. The C_2_ and C_3_ hydroxyl groups of CD form hydrogen bonds between adjacent glucose units. On the other hand, the C_6_-OH group presents free rotation. The adsorption of water molecules is faster in *β*CD than in *α*CD and *γ*CD owing to their different number of glucose units [10]. The crystalline structure (channel-type, cage-type, etc.) of CD complexes has also been studied. For example, the crystalline structure of *α*CD/polyethylene glycol complex has been reported to present a channel-type arrangement, while *β*CD/propylene glycol displays a channel-type (head-to-head or head-to-tail) or cage-type structure, and *γ*CD/salicylic acid or *γ*CD/flurbiprofen complexes exhibit channel-type (tetragonal, hexagonal, and monoclinic) arrangements [11–14]. The van der Waals forces and hydrogen bonds in the crystal structure of CD complexes have been reported to vary with the moisture and inclusion conditions [13]. In addition, a variety of methods, such as ground-mixing, freeze drying, and coprecipitation, are available for the preparation of inclusion complexes [15, 16]. The preparation of inclusion complexes using ground mixtures (GMs) is easy by exploiting mechanochemical effects. Inclusion complexes with molar ratios of PP/*α*CD = 1/2, PP/*β*CD = 1/1, and PP/*γ*CD = 1/1 prepared by such ground-mixing methods were found to exhibit different dissolution behavior in distilled water [17, 18]. However, to date, the factors contributing to the molecular conformation of PP in PP/CD complexes have not been evaluated in detail.

Solid-state fluorescence spectroscopy is a well-known method for the evaluation of the inclusion behavior of guest components [19]. Such measurements allow the elucidation of the molecular conformation of a guest molecule inside the CD cavity, either as a monomer or excited dimer. For example, changes in molecular morphology due to interaction of 2,5-diphenyloxazole/*γ*CD complexes have been evaluated by solid fluorescence measurement [20]. Therefore, solid-state fluorescence measurements are important for understanding the fluorescence properties that are closely related to conformation and sequence of molecules. However, to date, the molecular behavior of PP in the solid state has not been comprehensively evaluated. Therefore, in this study, the molecular behavior of PP in different PP/CD inclusion complexes (humidified, heated, and humidified-heated samples) was evaluated by detailed solid-state fluorescence measurements.

## 2. Materials and Methods

### 2.1. Materials

**Figure 1:**
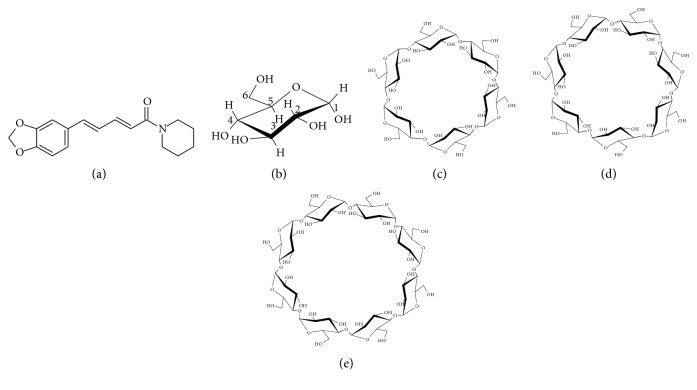
Chemical structure of (a) PP, (b) glucose, (c) *α*CD, (d) *β*CD, and (e) *γ*CD.

PP was purchased from FUJIFILM Wako Pure Chemical Co., Ltd. *α*CD, *β*CD, and *γ*CD were kindly donated by CycloChem Co., Ltd. (Tokyo, Japan), and used after storage at 40°C with a relative humidity (RH) of 82% for seven days. The humidity was controlled to obtain *α*CD 6.6-hydrate, *β*CD 10.5-hydrate, and *γ*CD 12-hydrate (Figure 1). All the other chemicals were used as received from FUJIFILM Wako Pure Chemical Co., Ltd.

### 2.2. Preparation of Inclusion Complexes

The molar ratio of PP/CD inclusion complexes has been previously reported to be PP/*α*CD = 1/2, PP/*β*CD = 1/1, and PP/*γ*CD = 1/1, as obtained by the ground mixture method [17, 18]. Thus, PP/CD complexes were prepared at those optimal inclusion molar ratios. GMs were prepared by grounding the samples (1.0 g) for 60 min using a vibration rod mill (TI-500ET, CMT Co.) at molar ratios of PP/*α*CD 6.6-hydrate = 1/2, PP/*β*CD 10.5-hydrate = 1/1, and PP/*γ*CD 12-hydrate = 1/1. Humidified GM (PP/CD) samples were prepared by humidifying (40°C, RH = 82%) the GMs for one week. Also, heated GM (PP/CD) samples were prepared by drying the GMs for 24 h at 105°C under vacuum (100 mbar abs.). Using a vacuum sample drying apparatus (VSD-95, ISHII SHOTEN Co.) humidified-heated GMs were prepared by humidifying (40°C, RH = 82%) the samples for one week, followed by drying for 24 h at 105°C under vacuum. Such humidified, heated, and humidified-heated GM (PP/CD) samples were prepared to evaluate the inclusion mechanisms in great detail.

### 2.3. Thermogravimetry (TG) Measurements

The thermal behavior of PP, CDs, and GMs was investigated by Thermo Gravimetry Analyzer (Thermo Plus Evo, Rigaku) under N_2_ gas flow rate of 200 mL/min and heating rate of 5°C/min. The samples (~5 mg) were placed in aluminum pans.

### 2.4. Solid-State Fluorescence Measurements

Fluorescence spectra in the solid state were recorded on a fluorescence spectrophotometer (RF-5300pc, Shimadzu). The measurement conditions were an excitation wavelength of 360 nm, excitation and fluorescence bandwidth of 5 nm, and measurement range from 380 to 600 nm.

### 2.5. Powder X-Ray Diffraction (PXRD) Measurements

The changes in the crystal diffraction pattern of the samples were observed using a powder X-ray diffractometer (MiniFlex II, Rigaku). The irradiation source was a Cu wire. The diffraction intensity was measured with NaI scintillation counter. Measurements were carried out at a voltage of 30 kV, current of 15 mA, scan range of 2*θ* = 3–35°, and scan rate of 4°/min.

### 2.6. Near-Infrared (NIR) Absorption Spectroscopy Measurements

NIR absorption spectra were recorded using a Fourier transform NIR spectrophotometer (NIRFlex N-500, Buchi). The measurement wavelengths and wavenumbers ranged from 1000 to 2500 nm and from 10000 to 4000 cm^−1^, respectively. The resolution was 8 cm^−1^ at a measurement temperature of 25°C. The second derivatives of the spectra were calculated.

## 3. Results and Discussion

### 3.1. Evaluation of Thermal Decomposition

**Figure 2:**
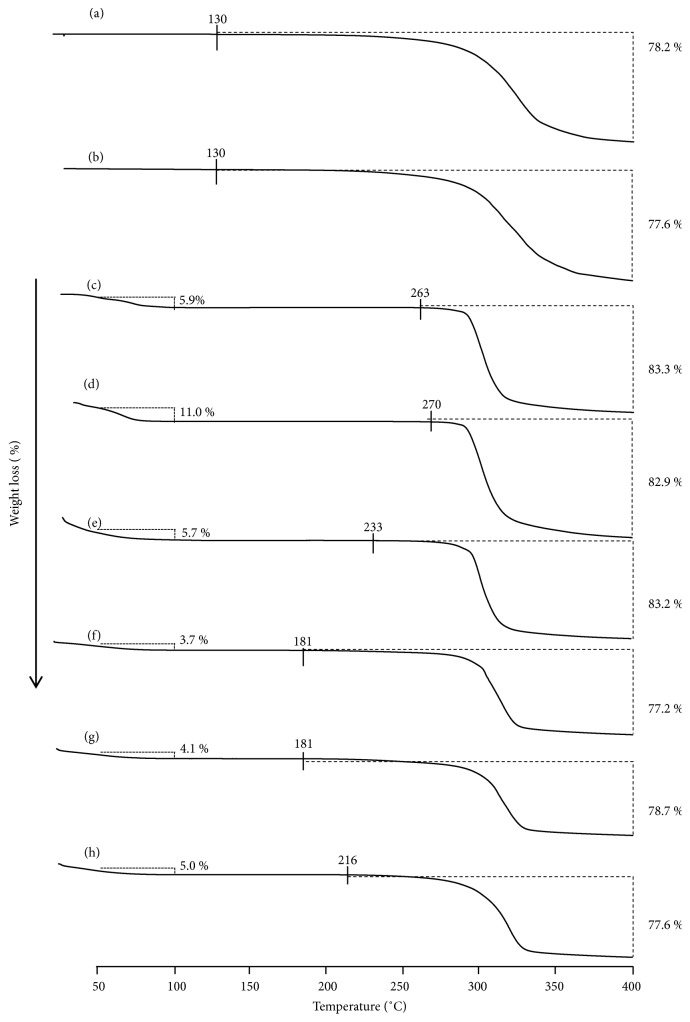
TG curves of (a) unprocessed PP, (b) PP ground, (c) *α*CD 6.6-hydrate, (d) *β*CD 10.5-hydrate, (e) *γ*CD 12-hydrate, (f) GM (PP/*α*CD = 1/2), (g) GM (PP/*β*CD = 1/1), and (h) GM (PP/*γ*CD = 1/1).

TG measurements were performed to evaluate the thermal stability and weight losses of PP upon complex formation (Figure 2). The weight loss of free PP was observed at ~130°C. In addition, the weight loss for GM (PP/*α*CD = 1/2), GM (PP/*β*CD = 1/1), and GM (PP/*γ*CD = 1/1) over the 50–100°C range was determined to be 3.7%, 4.1%, and 5.0%, respectively. The water loss from CD has been reported to occur up to 110°C [21]. Therefore, said weight losses in the inclusion complexes were concluded to arise from evaporation of the adsorbed water on the CDs. The weight of GM (PP/*α*CD = 1/2) decreased by 77.2% between 181 and 400°C, while that of GM (PP/*β*CD = 1/1) decreased by 78.7% in the same range. The weight of GM (PP/*γ*CD = 1/1) dropped by 77.6% between 216 and 400°C. It has been reported that the decomposition temperature of the guest component increases when a complex is formed [22]. The PP weight losses for the PP/CD complexes were observed at temperatures higher than that for free PP (130°C). Since the decomposition temperature of GM (PP/*γ*CD = 1/1) is higher than those of GM (PP/*α*CD = 1/2) and GM (PP/*β*CD = 1/1), it is suggested that GM (PP/*γ*CD = 1/1) is able to suppress more effectively the decomposition of PP.

### 3.2. Evaluation of the Steric Behavior of Molecules

**Figure 3:**
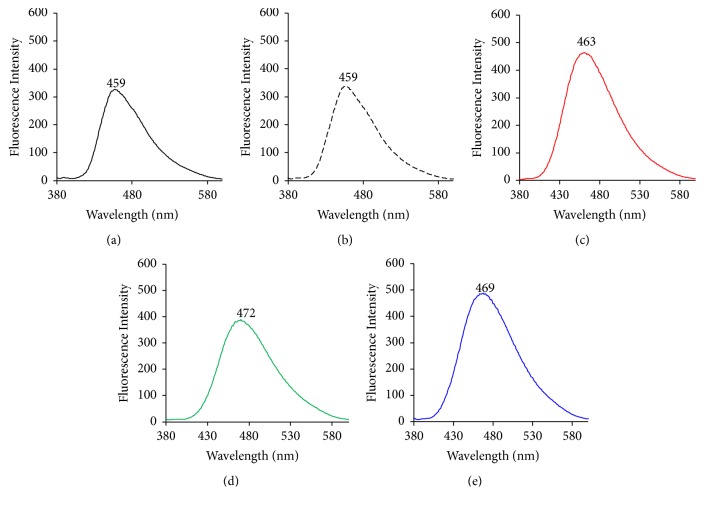
Emission spectra of unprocessed and ground mixtures of PP with different CDs. *λ*_ex_ = 360 nm: (a) unprocessed PP, (b) PP ground, (c) PP/*α*CD, (d) PP/*β*CD, and (e) PP/*γ*CD.

Depending on the molecular orientation of molecules in the solid state, the fluorescence wavelength is shifted by the energy difference from the excited state to the ground state [23]. There is a report that the fluorescence intensity decreases as the intermolecular interaction increases [24]. In addition, the oscillator strength affects the fluorescence intensity [25]. The greater the vibration and rotational motion of the molecule are, the more the energy of the excited electrons is consumed, so the fluorescence intensity decreases. That is, the solid-state fluorescence measurement is important for understanding the fluorescence characteristics closely related to the conformation and arrangement of molecules. Therefore, solid-state fluorescence measurements were carried out to determine whether the fluorescence wavelength would change with the different molecular behavior of PP. The fluorescence maximum of PP and PP ground was observed at 459 nm (Figure 3). On the other hand, the fluorescence maxima of the inclusion complexes GMs (PP/*α*CD = 1/2, PP/*β*CD = 1/1, and PP/*γ*CD = 1/1) were observed at 463, 472, and 469 nm, which are shifted to longer wavelengths compared to that of PP alone (Figures 3(c), 3(d), and 3(e)). Since the fluorescence maximum of PP shifts to longer wavelengths, it can be concluded that the electron resonance state (intramolecular charge) of PP changes upon interaction with the CD molecules [26]. According to the PP single crystal data reported by Pfund et al. (2015), the PP electrons resonate from the amide group to the ether group of the methylenedioxyphenyl moiety. Moreover, increased bond rotation around the amide or carbonyl group has been reported as the resonance state changes [27]. That is, the electrons donated by the nitrogen of PP are not transferred to the ether group, and an energy change occurs. Furthermore, the intermolecular interactions between adjacent molecules are suppressed and the fluorescence intensity increases upon changing into a molecular arrangement in which the overlap of *π*-conjugate planes is small.

**Figure 4:**
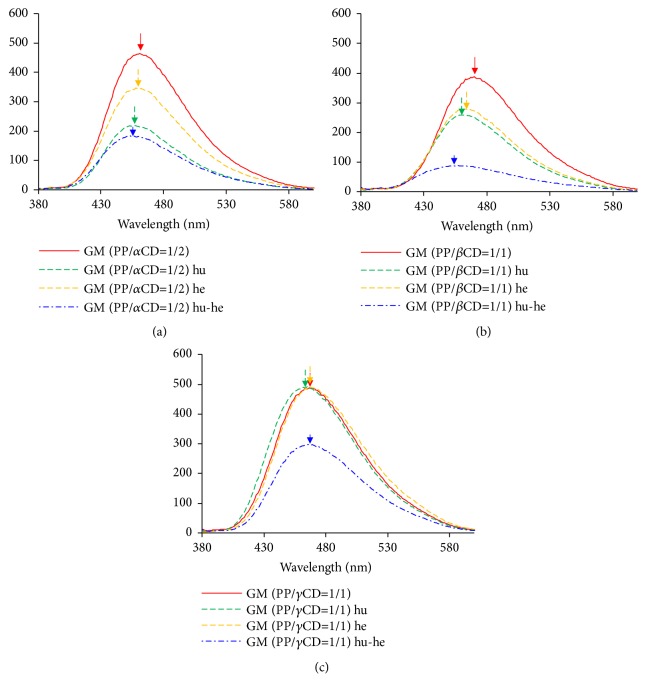
Changes in the emission spectra of PP/CD complex systems (*λ*_ex_ = 360 nm): (a) PP/*α*CD, (b) PP/*β*CD, and (c) PP/*γ*CD. Humidified: hu; heated: he; humidified-heated: hu-he.

In the *α*CD systems, the fluorescence maxima of the GM (PP/*α*CD = 1/2) and heated GM (PP/*α*CD = 1/2) samples were observed at 463 and 457 nm, respectively. On the other hand, the humidified and humidified-heated GM (PP/*α*CD = 1/2) samples exhibited maxima at 454 and 455 nm, respectively, which are shifted to shorter wavelengths compared to that of GM (PP/*α*CD = 1/2) (Figure 4(a)). In the fluorescence spectra of GM (PP/*α*CD = 1/2), humidified GM (PP/*α*CD = 1/2), and humidified-heated GM (PP/*α*CD = 1/2), the donation of the nitrogen group of PP changes upon coordination of water molecules, which contributes to a fluorescence change for the complexes with *α*CD.

In the *β*CD systems, the fluorescence maxima of GM (PP/*β*CD = 1/1) and heated GM (PP/*β*CD = 1/1) are observed at 472 and 462 nm, respectively. In addition, the humidified and humidified-heated GM (PP/*β*CD = 1/1) samples exhibit maxima at 460 and 455 nm, respectively, which are shifted to shorter wavelengths relative to that of the untreated GM (Figure 4(b)). It was thus concluded that the molecular behavior of PP in GM (PP/*β*CD = 1/1) and humidified GM (PP/*β*CD = 1/1) contributes to fluorescence quenching. In the fluorescence spectra of the untreated, humidified, and humidified-heated GM (PP/*β*CD = 1/1) samples, the donation of the nitrogen group of PP changes upon coordination of water molecules, contributing to changes in the fluorescence properties of the *β*CD complexes.

In the *γ*CD systems, the fluorescence maxima of the untreated, heated, and humidified-heated GM (PP/*γ*CD = 1/1) samples were observed at 469 nm. On the other hand, humidified GM (PP/*γ*CD = 1/1) exhibited a maximum at 465 nm, slightly shifted toward shorter wavelengths relative to the value for the untreated GM (Figure 4(c)). Since the maximum fluorescence wavelength of GM (PP/*γ*CD = 1/1) is similar to that of humidified and humidified-heated GM (PP/*γ*CD = 1/1) samples, the molecular behavior is also similar. Therefore, it can be speculated that the conformation of PP molecules in humidified crystalline PP/CD inclusion complexes changes depending on the size of the CD cavity, where the coordination of water also contributes to the overall effect.

### 3.3. Examination of the Crystalline State

PXRD measurements confirmed the changes in the crystalline state of PP/CD complexes upon coordination of water molecules. The pattern of pure PP exhibits characteristic diffraction peaks at 2*θ* = 14.7° and 25.6°. In the TG measurement, since CD included PP, no weight loss of PP's residual crystals was confirmed in various complexes. it is reported that the melting point of PP alone disappears due to the formation of PP/CDs complex. Also, the PXRD pattern of PP and PP ground has been reported to be the same diffraction peak [17, 18]. It has been reported that PP/*α*CD, PP/*β*CD, and PP/*γ*CD inclusion complexes prepared by ground-mixing afford a halo pattern [17, 18]. The heated, humidified, and humidified-heated GM (PP/CD) samples were used to identify changes in the diffraction patterns of the complexes.

**Scheme 1:**
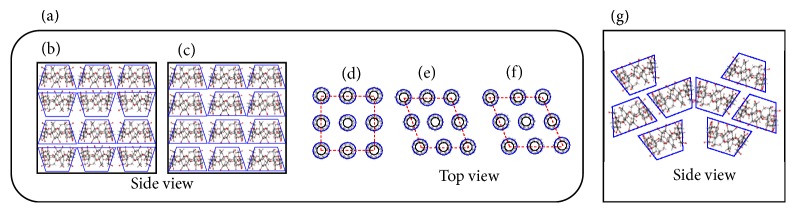
Structural view of different crystalline arrangements: (a) channel-type, (b) head-to-head, (c) head-to-tail, (d) tetragonal, (e) monoclinic, and (f) hexagonal; (g) cage-type.

**Figure 5:**
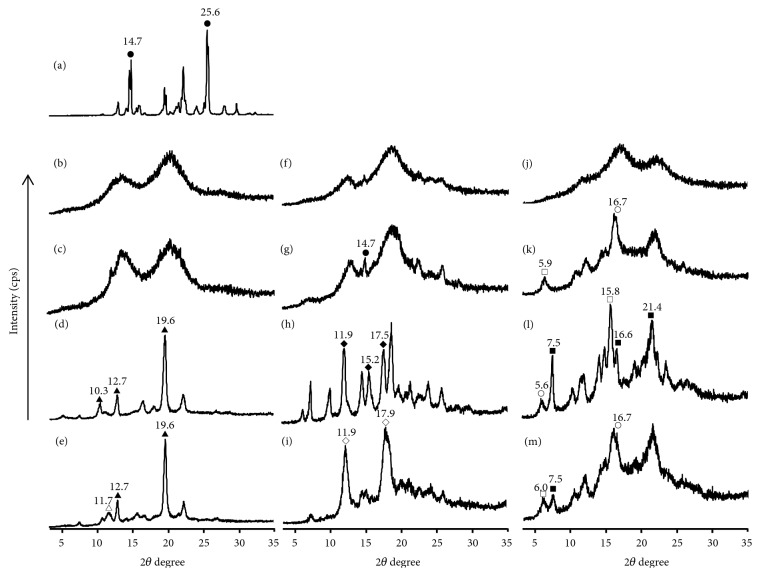
Changes in the PXRD patterns of PP/CD complex systems: (a) unprocessed PP; (b) GM (PP/*α*CD = 1/2), (c) heated GM (PP/*α*CD = 1/2), (d) humidified GM (PP/*α*CD = 1/2), and (e) humidified-heated GM (PP/*α*CD = 1/2); (f) GM (PP/*β*CD = 1/1), (g) heated GM (PP/*β*CD = 1/1), (h) humidified GM (PP/*β*CD = 1/1), and (i) humidified-heated GM (PP/*β*CD = 1/1); (j) GM (PP/*γ*CD = 1/1), (k) heated GM (PP/*γ*CD = 1/1), (l) humidified GM (PP/*γ*CD = 1/1), and (m) humidified-heated GM (PP/*γ*CD = 1/1). ●: PP; ▲: channel (wet); △: channel (dried); ◆: head-to-tail; *◇*: cage-type; ○: monoclinic; □: hexagonal; ■: tetragonal.

In the *α*CD system, the diffraction profiles of the untreated and heated GM (PP/*α*CD = 1/2) systems exhibit a halo pattern (Figures 5(b) and 5(c)), while humidified GM (PP/*α*CD = 1/2) presents diffraction peaks at 2*θ* = 10.3°, 12.7°, and 19.6° suggesting a channel-type arrangement (Figure 5(d), Scheme 1). The humidified-heated GM (PP/*α*CD = 1/2) sample exhibits the characteristic diffraction peak of a dried channel-type structure (where both head-to-head and head-to-tail arrangements are mixed) at 2*θ* = 11.7° (Figure 5(e), Schemes 1(b) and 1(c)) [12]. No peaks corresponding to PP alone are observed in the humidified GM (PP/*α*CD = 1/2) and humidified-heated GM (PP/*α*CD = 1/2) pattern. It was suggested that there were few residual crystals of PP (Figures 5(d) and 5(e)). It was thus concluded that the fluorescence spectra of the humidified and humidified-heated GM (PP/*α*CD = 1/2) samples show similar features of a channel-type structure despite the different coordination of water molecules.

In the *β*CD system, the diffraction pattern of GM (PP/*β*CD = 1/1) presents a halo profile (Figure 5(f)). The pattern of the heated GM (PP/*β*CD = 1/1) sample shows the characteristic peak of PP at 2*θ* = 14.7° (Figure 5(g)). It is suggested that the PP/*β*CD complex dissociates into PP and *β*CD to some extent under heating. Such PP peak is not observed for the humidified GM (PP/*β*CD = 1/1) sample; however, the diffraction pattern shows the characteristic diffraction peaks of head-to-tail *β*CD at 2*θ* = 11.9°, 15.2°, and 17.5° (Figure 5(h)) [13, 14]. GM (PP/*β*CD = 1/1) changed from an amorphous state to a regular crystal sequence of head-to-tail by water molecules coordinating. Humidified GM (PP/*β*CD = 1/1) was shifted to the short wavelength compared with GM (PP/*β*CD = 1/1) (Figure 4(b)). This change suggests that the nitrogen donating property and motility of PP influence the molecular morphology and behavior because the molecular arrangement is different. The diffraction peak of CDs is influenced by the coordination of water molecule. It contributed to the interaction with PP, possibly affecting fluorescence. Furthermore, the humidified-heated GM (PP/*β*CD = 1/1) sample exhibits the characteristic diffraction peaks of a cage-type arrangement at 2*θ* = 11.9° and 17.9° (Figure 5(i)). That is, the humidified GM (PP/*β*CD = 1/1) sample changes from a head-to-tail structure to a cage-type one under heating. No peaks corresponding to PP alone are observed in the humidified GM (PP/*β*CD = 1/1) and humidified-heated GM (PP/*β*CD = 1/1) pattern. It was suggested that there were few residual crystals of PP. The humidified GM (PP/*β*CD = 1/1) sample is therefore concluded to involve an inclusion structure that readily changes upon adsorption of water molecules in the crystal.

In the *γ*CD system, the diffraction profile of GM (PP/*γ*CD = 1/1) shows a halo pattern (Figure 5(j)). No peaks corresponding to PP alone are observed in the heated GM (PP/*γ*CD = 1/1) pattern, but diffraction peaks corresponding to mixed hexagonal-type (2*θ* = 5.9°) and monoclinic-type (2*θ* = 16.7°) structures (Figure 5(k)) are discerned [15]. The characteristic diffraction pattern of the inclusion complex was thus confirmed. The humidified GM (PP/*γ*CD = 1/1) sample was found to exhibit the characteristic diffraction peaks for tetragonal-type (2*θ* = 7.5°, 16.6°, and 21.4°), monoclinic-type (2*θ* = 5.6°), and hexagonal-type (2*θ* = 15.8°) arrangements (Scheme 1(f), Figure 5(l)). Furthermore, the pattern of humidified-heat GM (PP/*γ*CD = 1/1) shows the characteristic diffraction peaks of hexagonal-type (2*θ* = 6.0°), tetragonal-type (2*θ* = 7.5°), and monoclinic-type (2*θ* = 16.7°) structures (Figure 5(m)). Tetragonal-type of *γ*CD complexes has been reported to change into hexagonal or monoclinic-type structures by decreasing crystalline water [15]. However, the diffraction peaks for the heated, humidified, and humidified-heated GM (PP/*γ*CD = 1/1) samples were those corresponding to mixed tetragonal-type, hexagonal-type, and monoclinic-type structures. No peaks corresponding to PP alone are observed in the humidified (PP/*γ*CD = 1/1) and humidified-heated (PP/*γ*CD = 1/1) pattern. Even if PP residual crystals remain, they are very few. The *γ*CD complex exists in a disordered and distorted state due to the influence of the PP conformation, and it is therefore expected that sublimation of the crystallized water located between the *γ*CD molecules and the subsequent progression from amorphous to crystalline state are difficult.

### 3.4. Examination of Intermolecular Interactions in Different Inclusion Complexes

**Figure 6:**
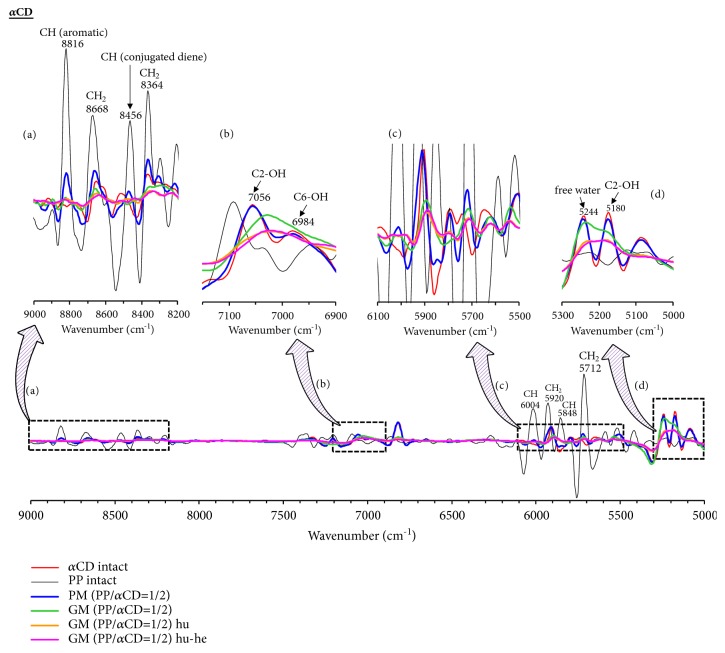
Second-derivative NIR absorption spectra of PP/*α*CD systems: (a) 9000–8200 cm^−1^; (b) 7150–6900 cm^−1^; (c) 6100–5500 cm^−1^; and (d) 5300–5000 cm^−1^. Humidified: hu; heated: he; humidified-heated: hu-he.

**Figure 7:**
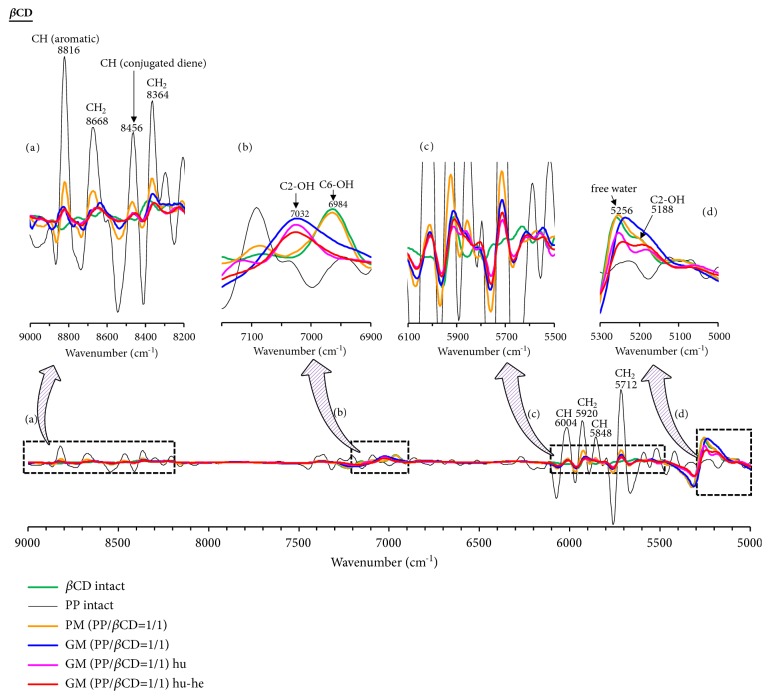
Second-derivative NIR absorption spectra of PP/*β*CD systems: (a) 9000–8200 cm^−1^; (b) 7150–6900 cm^−1^; (c) 6100–5500 cm^−1^; and (d) 5300–5000 cm^−1^. Humidified: hu; heated: he; humidified-heated: hu-he.

**Figure 8:**
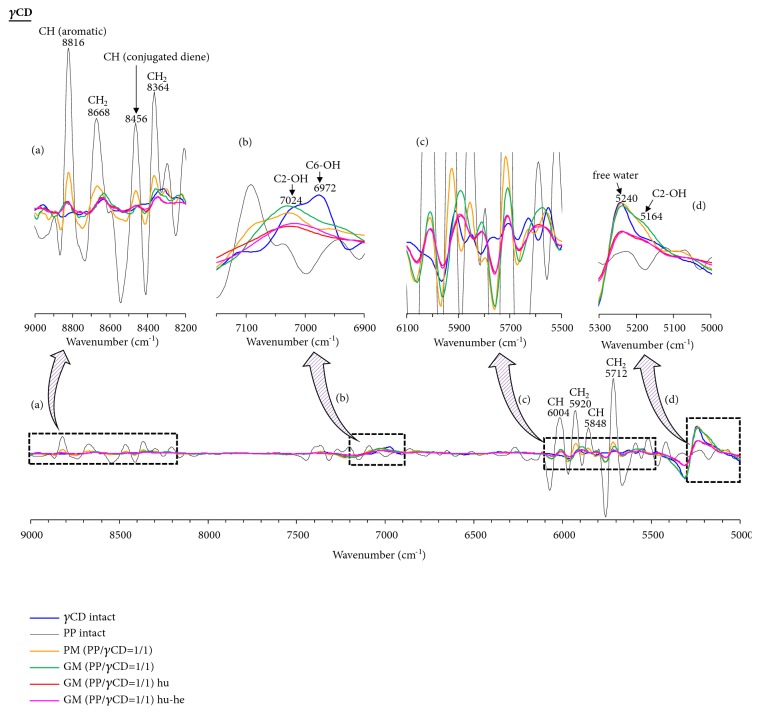
Second-derivative NIR absorption spectra of PP/*γ*CD systems: (a) 9000–8200 cm^−1^; (b) 7150–6900 cm^−1^; (c) 6100–5500 cm^−1^; and (d) 5300–5000 cm^−1^. Humidified: hu; heated: he; humidified-heated: hu-he.

In the TG measurements, the weight loss of PP was found to be suppressed upon formation of the PP/CD complexes. In the PXRD measurements, the crystal diffraction pattern of the PP/CD complexes was found to depend on the relative humidity and heating conditions. Thus, NIR spectroscopy would be the optimal method to evaluate the presence of free water in the solid state without interference from the included water molecules, where the stretching vibrations of OH and CH groups can be easily evaluated [28]. The CH_2_ bands of PP were observed at 5712, 5920, 8364, and 8668 cm^−1^. In addition, the CH bands of the conjugated double bond of the geometric isomer of PP were also found at 5848, 6004, and 8456 cm^−1^. Finally, the band corresponding to the aromatic CH moiety of PP was observed at 8816 cm ^−1^ (Figures 6–8).

**Scheme 2:**
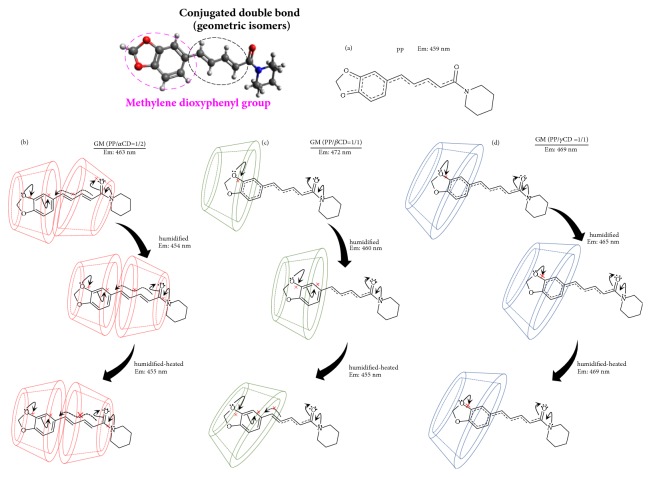
Changes in the molecular state of PP upon ground-mixing with different CDs: (a) PP, (b) GM (PP/*α*CD = 1/2), (c) GM (PP/*β*CD = 1/1), and (d) GM (PP/*γ*CD = 1/1).

In the *α*CD system, the CH_2_ band (8364 and 8668 cm^−1^) of GM (PP/*α*CD = 1/2) was shifted to lower wavenumbers (8352 and 8648 cm^−1^) and the molecular vibrations of the conjugated diene and aromatic CH (8456 and 8816 cm^−1^) were decreased compared to those of PP alone. Thus, the molecular behavior of PP was confirmed to change upon complex formation (Figure 6(a)). In addition, the CH_2_ vibration bands (8364 and 8668 cm^−1^) of GM (PP/*α*CD = 1/2) decreased and those corresponding to conjugated diene and aromatic CH moieties (8456 and 8816 cm^−1^) were broader compared to those of PP alone. The mobility of the PP molecule therefore decreases upon changing from amorphous GM (PP/*α*CD = 1/2) to the columnar structure of humidified GM (PP/*α*CD = 1/2). It was concluded that the changes in the mobility of PP are influenced by distance of the *α*CD molecules allowing the formation of hydrogen bonds. Furthermore, the CH_2_ band (8456 cm^−1^) of the conjugated diene of GM (PP/*α*CD = 1/2) shifts to a lower wavenumber (8428 cm^−1^) compared to that of humidified GM (PP/*α*CD = 1/2) with a head-to-head crystal structure. The humidified-heated GM (PP/*α*CD = 1/2) sample exhibits the characteristic diffraction pattern of a dried channel-type structure (mixed head-to-head and head-to-tail) (Figures 5(d) and 5(e)). In the NIR measurements, the CH_2_ and aromatic CH bands of humidified and humidified-heated GM (PP/*α*CD = 1/2) were broader than those of GM (PP/*α*CD = 1/2) (Figure 6(a)). It is suggested that the molecular mobility of the conjugated diene of PP in humidified-heated GM (PP/*α*CD = 1/2) decreases owing to a reduction of the hydrogen bond length as *α*CD molecules become closer, fixing the PP molecules. That is, the resonance states of humidified and humidified-heated GM (PP/*α*CD = 1/2) change due to the interaction between the methylenedioxyphenyl group (aromatic ring moiety) of PP and *α*CD, and the fluorescence wavelength of PP shifts to shorter wavelengths (Scheme 2(b)).

In the *β*CD system, the CH_2_ bands (8364 and 8668 cm^−1^) of the conjugated diene of GM (PP/*β*CD = 1/1) shift to lower wavenumbers (8344 and 8652 cm^−1^) compared to those of PP alone. In addition, the absorption band (8816 cm^−1^) of the aromatic CH of GM (PP/*β*CD = 1/1) decreases compared to that of PP alone. The molecular behavior of PP was observed to change upon complex formation (Figures 7(a) and 7(c)). The absorption intensity of the CH_2_ bands (5712 and 5920 cm^−1^) of humidified GM (PP/*β*CD = 1/1) decreases, and the aromatic CH band (8816 cm^−1^) shifts to a higher wavenumber (8832 cm^−1^) relative to that of PP alone. The molecular mobility of PP is reduced upon changing from amorphous GM (PP/*β*CD = 1/1) to the columnar structure of humidified GM (PP/*β*CD = 1/1). It is concluded that the molecular mobility of PP decreases due to the formation of hydrogen bonding between *β*CD and changes in the van der Waals forces. In addition, the CH band (5848 cm^−1^) corresponding to the conjugated diene of humidified-heated GM (PP/*β*CD = 1/1) was broader than that of humidified GM (PP/*β*CD = 1/1). Since the humidified-heated GM (PP/*β*CD = 1/1) sample exhibits the crystal diffraction pattern of a cage-type structure, it is considered that the distance between *β*CD units is reduced compared to that in humidified GM (PP/*β*CD = 1/1) and that the molecular mobility of the conjugated diene of PP is subsequently also reduced. From the solid-state fluorescence measurements, the resonance state of the humidified and humidified-heated GM (PP/*β*CD = 1/1) samples is concluded to change under the interaction of the methylenedioxyphenyl CH band (5848 cm^−1^) of PP, which shifts to shorter wavelengths. Furthermore, when the CH band (5848 cm^−1^) of the conjugated diene of humidified-heated GM (PP/*β*CD = 1/1) broadens, its fluorescence maximum wavelength shifts toward those of humidified and humidified-heated GM (PP/*α*CD = 1/2). That is, the molecular mobility of the methylenedioxyphenyl group and conjugated diene CH is strongly suppressed upon PP interaction with the CD; the said PP molecular behavior contributes to fluorescence quenching (Scheme 2(c)).

In the *γ*CD system, the CH_2_ bands (8364 and 8668 cm^−1^) and the conjugated diene CH bands (5848 and 8456 cm^−1^) of GM (PP/*γ*CD = 1/1) broaden and shift to lower wavenumbers (8448 cm^−1^) compared to those of unprocessed PP (Figures 8(a) and 8(c)). Moreover, the aromatic CH band (8816 cm^−1^) of GM (PP/*γ*CD = 1/1) shifts to higher wavenumbers (8828 cm^−1^) (Figure 8(a)). Suppression of the molecular mobility of PP thus occurs upon complex formation. The absorption intensity of the CH_2_ bands (5712, 8364, and 8668 cm^−1^) of humidified GM (PP/*β*CD = 1/1) decreases, and the aromatic CH band (8816 cm^−1^) shifts to a higher wavenumber relative to that of PP alone (Figures 8(a) and 8(c)). Changes in the molecular mobility of PP arise upon moving from the amorphous structure of GM (PP/*γ*CD = 1/1) to the columnar arrangement of humidified GM (PP/*γ*CD = 1/1). It is concluded that the changes in the molecular mobility of PP are influenced by the formation of hydrogen bonding between *γ*CD molecules and changes in the van der Waals forces. The interaction between PP methyl group and CD hydroxyl group in humidified-heated GM (PP/*γ*CD = 1/1) is similar to that observed in humidified GM (PP/*γ*CD = 1/1). Therefore, it was confirmed that, in *γ*CD with a large annular size, the molecular behavior of PP in the channel-type inclusion structure is not remarkably suppressed (Scheme 2(d)). The molecular mobility of PP is reduced upon changing from the amorphous GM (PP/CD) systems to the columnar structures of the humidified GM (PP/CD) samples. The mobility of PP does not change even with changes in the crystal structure of humidified-heated GM (PP/*γ*CD), which is a dried humidified GM (PP/*γ*CD) sample. On the other hand, the motility of PP was confirmed to easily change in humidified-heated GM (PP/*α*CD = 1/2) and humidified-heated GM (PP/*β*CD = 1/1). Therefore, it was confirmed that the molecular behavior of PP varies depending on the ring size. It is reported that the stability constant of PP/CD systems increases in the order *α*CD < *β*CD < *γ*CD [18]. The stability constant is a representation of the distance between the hydroxyl group of the ether group of PP and the cavity diameter of CD and the ability to form hydrogen bond. The molecular behavior of PP in these crystal structures is shown to be fixed in the cases of *α*CD and *β*CD.

### 3.5. Influence of CD-Containing Water Molecules and Evaluation of OH Group Interactions

The influence of the water molecules adsorbed on the CDs and the interactions of the OH groups were evaluated by NIR spectroscopy. Since PP bears no OH groups and is a lipophilic drug, bands corresponding to water molecules and hydroxyl groups were not observed in its spectrum in the ranges of 5300–5000 and 7200–6900 cm^−1^ (Figures 6–8). In the case of *α*CD, C_2_-OH (5180, 7056 cm^−1^), C_6_-OH (6984 cm^−1^), and free water (5244 cm^−1^) bands were observed (Figures 6(b) and 6(d)). The C_2_-OH of *β*CD tends to adsorb water molecules, for which a band is observed at 5188 cm^−1^ (Figure 7(d)). In contrast, it was difficult to evaluate the C_2_-OH bands in the 7200–6900 cm^−1^ region. In addition, bands for the C_6_-OH moiety (6960 cm^−1^) and free water (5256 cm^−1^) in *β*CD were observed. *γ*CD has a noncoplanar and more flexible structure and the OH groups can be easily approached by water molecules [29]. Therefore, the C_2_-OH (5164 and 7024 cm^−1^), C_6_-OH (6972 cm^−1^), and free water (5240 cm^−1^) bands are all observed in the spectrum of *γ*CD (Figures 8(b) and 8(d)).

In the *α*CD system, the free water bands for GM (PP/*α*CD = 1/2) decrease compared to those of *α*CD alone (Figure 6(d)). The bands for C_2_-OH (5180 and 7056 cm^−1^) and C_6_-OH (6984 cm^−1^) were broadened and that of C_2_-OH (7056 cm^−1^) was shifted to lower wavenumbers (7032 cm^−1^). It has been reported that the four units of glucose of *α*CD present hydrogen bonding between the C_2_-OH and C_3_-OH moieties. The bands corresponding to C_2_-OH, C_6_-OH, and free water in the case of humidified GM (PP/*α*CD = 1/2) were broader than those of GM (PP/*α*CD = 1/2) (Figures 6(b) and 6(d)). It is suggested that *α*CD forms a hydrogen bonding network with other *α*CD molecules in addition to PP. The C_2_-OH band (7056 cm^−1^) of humidified-heated GM (PP/*α*CD = 1/2) was broader and slightly shifted to lower wavenumbers compared to that of humidified GM (PP/*α*CD = 1/2). Therefore, it is suggested that the distance between *α*CD molecules decreases and stronger hydrogen bonds are formed with the decreasing number of water molecules.

In the *β*CD system, the band corresponding to free water in the case of GM (PP/*β*CD = 1/1) decreased compared to that for *β*CD alone. Furthermore, the C_2_-OH band was observed at 7032 cm^−1^ and that of C_6_-OH disappeared at 6960 cm^−1^ (Figures 7(b) and 7(d)). *β*CD presents strong hydrogen bonding between the C_2_-OH and C_3_-OH moieties of glucose, and the absorption intensity decreases upon adsorption of water molecules, reducing the molecular mobility of *β*CD. That is, the adsorption of water molecules on the C_2_-OH moieties of GM (PP/*β*CD = 1/1) decreases upon inclusion of PP in the *β*CD cavity. The free water band (5256 cm^−1^) of humidified GM (PP/*β*CD = 1/1) decreases and the C_2_-OH band (7032 cm^−1^) becomes sharper compared to those of GM (PP/*β*CD = 1/1). Therefore, the reduction of the number of water molecules and C_2_-OH band suggests the formation of hydrogen bonding between PP and *β*CD and between *β*CD molecules. In addition, the free water band (5256 cm^−1^) of humidified-heated GM (PP/*β*CD = 1/1) decreases and the C_2_-OH band (7032 cm^−1^) shifts to higher wavenumbers (7028 cm^−1^) relative to those of humidified GM (PP/*β*CD = 1/1). The cage-type structure, which is the crystal structure of *β*CD complexes, has been reported to exhibit strong van der Waals forces and weak hydrogen bonding, and the head-to-tail column-type has been associated with weak van der Waals forces and moderate hydrogen bonding [13]. Therefore, the C_2_-OH band of humidified-heated GM (PP/*β*CD = 1/1) shifts to higher wavenumbers due to the weakening of hydrogen bonding.

In the *γ*CD system, the C_6_-OH band (6972 cm^−1^) of GM (PP/*γ*CD = 1/1) broadens and shifts to lower wavenumbers, while the C_2_-OH band (5164, 7024 cm^−1^) broadens and shifts to higher wavenumbers (7040 cm^−1^) compared to those of *γ*CD alone (Figures 8(b) and 8(d)). It was inferred that the hydrogen bonding via the C_2_-OH moieties of GM (PP/*γ*CD = 1/1) decreases upon complex formation and that hydrogen bonding between C_6_-OH and PP is established. The humidified GM (PP/*γ*CD = 1/1) sample exhibits broadband for the C_6_-OH (6972 cm^−1^) and C_2_-OH (5164 and 7024 cm^−1^) moieties, while the free water band of *γ*CD decreases compared to that of GM (PP/*γ*CD = 1/1). That is, a hydrogen bond network is generated between *γ*CD molecules. The free water band of humidified-heated GM (PP/*γ*CD = 1/1) is similar to that of humidified GM (PP/*γ*CD = 1/1). However, the C_2_-OH band (7024 cm^−1^) shifts to lower wavenumbers (7020 cm^−1^) compared to that of humidified GM (PP/*γ*CD = 1/1), and so a difference in the molecular mobility is noted. Since the C_2_-OH band shifts to higher wavenumbers, it is inferred that the hydrogen bonding extent decreases due to changes in the crystal structure. The CH group (aromatic and conjugated diene) mobility in humidified GM (PP/*γ*CD = 1/1) is similar to that in humidified-heated GM (PP/*γ*CD = 1/1). However, it was confirmed that the C_2_-OH molecular behavior in humidified GM (PP/*γ*CD = 1/1) is different (Figure 8). Even when changing from GM (PP/*γ*CD = 1/1) to humidified GM (PP/*γ*CD = 1/1) and humidified-heated GM (PP/*γ*CD = 1/1), the motility of the aromatic ring CH of the methylenedioxyphenyl group of PP embedded in *γ*CD does not change. Moreover, even if the crystal structure of the *γ*CD complex changes, the molecular mobility of the aromatic CH of the methylenedioxyphenyl group of PP is not suppressed compared to that in the *α*CD and *β*CD complexes. The reason behind this lies in differences in the moisture adsorption and crystallization of *α*CD, *β*CD, and *γ*CD. The maximum fluorescence wavelengths of humidified and humidified-heated *α*CD and *β*CD inclusion complexes shift toward that of PP alone. The reason is that the molecular motion of the aromatic ring CH of the methylenedioxyphenyl group of PP is fixed inside *α*CD and *β*CD, contributing to changes in the electron resonance. Therefore, solid fluorescence and NIR measurements have demonstrated that the molecular behavior of PP is influenced by the presence or absence of water molecules in the CD structure.

## 4. Conclusions

By solid-state fluorescence measurements, it was demonstrated that the conformation of PP inside *α*, *β*, and *γ*CD is influenced by the resonance from the nitrogen group to the ether group upon coordination of water. Moreover, it was revealed that the *γ*CD complexes are disordered and distorted in nature due to the influence of the conformation of PP, resulting in a mixture of crystal structures, as compared to the systems with *α*CD and *β*CD. The molecular behavior of PP inside CD changes its fluorescence behavior by suppressing the motion of the aromatic CH center of the methylenedioxyphenyl group. Furthermore, molecular behavior of PP also varies with the coordination of water and the cavity size. Therefore, from the results of solid-state fluorescence and NIR measurements, it has been demonstrated that the transfer of electrons from the nitrogen group to the ether group depends on the mobility of the aromatic CH moiety of the methylenedioxyphenyl group. The detailed molecular behavior of PP in these complexes has thus been revealed.

## Data Availability

The data used to support the findings of this study are included within the article. Previously reported data were used to support this study and are available at DOI: 10.1155/2016/8723139 and DOI: 10.1208/s12249-017-0908-9. These prior studies are cited at relevant places within the text as [17, 18].

## Abbreviations

PP: Piperine
CD: Cyclodextrin
GM: Ground mixture
PM: Physical mixture
RH: Relative humidity
Humidified GM (PP/CD): GM (PP/CD) sample humidified (40°C, RH = 82%) for one week
Heated GM (PP/CD): GM (PP/CD) sample dried for 24 h at 105°C under vacuum
Humidified-heated GM (PP/CD): GM (PP/CD) sample humidified (40°C, RH = 82%) for one week, followed by drying for 24 h at 105°C under vacuum
C2-OH: Hydroxyl group attached to the carbon at position 2 of glucose
C6-OH: Hydroxyl group attached to the carbon at position 6 of glucose
TG: Thermogravimetry
PXRD: Powder X-ray diffraction
NIR spectroscopy: Near-infrared spectroscopy.
